# Prevalence of HLA-B27 in Patients with Ankylosing Spondylitis in Qatar

**DOI:** 10.1155/2012/860213

**Published:** 2012-04-04

**Authors:** M. H. Abdelrahman, S. Mahdy, I. A. Khanjar, A. M. Siam, H. A. Malallah, S. A. Al-Emadi, H. A. Sarakbi, M. Hammoudeh

**Affiliations:** Rheumatology Section, Department of Medicine, Hamad General Hospital, P.O. Box 3050, Doha, Qatar

## Abstract

*Background and Objectives*. The human leukocyte antigen HLA-B27 is a class 1 antigen of the major histocompatibility complex and is strongly associated with ankylosing spondylitis (AS). The purpose of the present study is to investigate the distribution of HLA-B27 in patients with AS of different ethnic groups in Qatar. *Design and Setting*. Study design was cross-sectional and the setting was rheumatology clinics of Hamad General Hospital in Qatar where most of ankylosing spondylitis patients are followed up. *Patients and Methods*. Patients with diagnosis of AS who met the New York modified criteria for AS were tested for HLA-B27. 119 patients were tested for HLA-B27: 66 Arabs, 52 Asians (Indians, Pakistanis, Bengalis, and Iranians), and one Western (Irish). *Results*. Of all the individuals, 82 were positive (69%) for HLA-B27. Among the Arabs, 49/66 were positive (74%). Among the Asians, 32/52 were positive (61%). Furthermore, Qatari patients (10 males and one female) 9 were positive (82%), 14/19 Jordanians/Palestinians were positive, and 9/10 (90%) Egyptians were positive. Among the Asians, 19/26 Indians were positive (73%), which was similar to the Arabs. *Conclusion*. HLA-B27 in our small group of Arabs is present in 74%. Comparison with other data will be presented in detail.

## 1. Introduction

The human leukocyte antigen (HLA-B27) is a class I antigen of the major histocompatibility complex, and it is strongly associated with ankylosing spondylitis and other related spondyloarthropathies (SpAs). It is present in only 8% of the general population worldwide [1]. In the Middle East, lower figures were reported from Arab countries, that is, United Arab Emirates (UAE) 0.5%, Saudi Arabia 2.6%, Kuwait 4%, Iraq 2.1%, Lebanon 1.4%, Tunisia 3.2%, and Syria 1.4% [2–8]. On the other hand, a remarkably higher percentage was found in Yemeni population (17%) [9].

In AS patients, HLA-B27 is present in 80–95% worldwide [1]. The prevalence of HLA-B27 among AS patients in the Arab world is generally lower than the worldwide figure, ranging from 56 to 84%: 84% in Iraq, 56% in UAE, 67% in Saudi Arabia, 58.6% in Egypt, 60% in Syria, and 73.4% in Iran [5, 9–12].

The prevalence of HLA-B27 among healthy persons and patients with AS in Qatar is unknown.

In this study we tested 119 of AS patients followed in rheumatology outpatient clinics of Hamad General Hospital in Qatar for the status of HLA-B27. This is the first study in Qatar to assess the prevalence of HLA-B27 among patients with AS who are residents of Qatar (locals and expatriates).

## 2. Patients and Methods

One hundred nineteen patients with diagnosis of AS who met the 1984 New York modified criteria for AS were tested for HLA-B27. There were 66 Arabs (55.5%), 52 Asians (43.7%), and one Western. Among the Arabs, 11 were Qataris (9.2%) (10 males and 1 female), 19 were Jordanians/Palestinians (15.9%), 10 were Egyptians (8.4%), and 26 were of other Arab nationalities. Among the Asians, 26 were Indians (21.8%) and 26 were of other Asian nationalities for example, Pakistanis, Bengalis, and Iranians.

Qatar is an Arab country, in the Middle East, occupying the small Qatar Peninsula on the northeasterly coast of the much larger Arabian Peninsula. The total Population of Qatar is 1,670,389 individuals, 1,270,968 males and 399,421 females [13]. Expatriates form the majority of Qatar residents, nearly 3/4th of the population.

## 3. Results

Of all the individuals (119), 82 were HLA-B27 positive (69%). Among the Arabs, 49/66 were positive (74%) and among the Asians 32/52 were positive (61%). Positive HLA-B27 was found in 9/11 Qataris (82%), 14/19 Jordanian/Palestinians (72%), 9/10 Egyptians (90%), and 19/26 Indians (73%) (Table 1).

## 4. Discussion

This study was conducted to assess the prevalence of HLA-B27 among patients with AS living in Qatar. The overall percentage was 69% and a higher prevalence was found among locals (82%). This prevalence in Qataris is close to the prevalence in the West and to the prevalence reported in Jordan of 75 and 81% and in Iraq of 84% [5, 14, 15].

Data from other Arabian Gulf countries shows that the prevalence of HLA-B27 is lower. In UAE, Al Attia found the percentage to be 56% among Arabs although none of them were locals and the study sample was rather small (28 pts.) [9]. It is worth mentioning that the percentage of HLA-B27 among Emirian Arabs is extremely low (0.5%) [2]. In Saudi Arabia, HLA-B27 was checked in 12/15 Arabs, locals comprised 6/15, and the percentage was only 67% [10]. In Kuwait, the percentage was higher (77.8%), but Kuwaitis were only 9 out of 58 [16]. In our study the percentage in 19 Jordanian patients was 72%, this was slightly lower than the findings in 2 Jordanian papers, 75% in the first paper with total of 20 patients tested and 81% in the second paper with total of 52 patients tested [14, 15]. Ten Egyptian patients were tested in our study and the result was positive in 9 of them (90%), which is still within the worldwide figure but much higher than the data from Egypt (58.6%) [11]. Our study included the Asian population also, and, among 26 Indians, 73% were HLA-B27 positive, this is close to the frequency reported among Indians in studies done in the Middle East and India [9, 17].

## 5. Conclusion

This is the first study to evaluate the prevalence of HLA-B27 among AS patients living in Qatar. The population studied includes locals, other Arabs, and Asians. The results suggest that prevalence of HLA-B27 among Qatari patients is similar to the prevalence seen in the West. There is a lack of knowledge about the percentage of HLA-B27 in healthy Qataris.

Further studies with larger number of patients and studies that look at the prevalence of AS in Qatar and at the prevalence of HLA-B27 in healthy Qataris are needed.

## Figures and Tables

**Table 1:** HLA-B27 distribution among patients with AS in Qatar.

Nationality	Number	+ve HLA-B27
Qataris	11	09 (82%)
Jordanians/Palestinians	19	14 (72%)
Egyptians	10	09 (90%)
Iraqis	05	04 (80%)
Omanis	04	03 (75%)
Sudanese	04	03 (75%)
Syrians	04	02 (50%)
Lebanese	04	02 (50%)
Yemenis	03	02 (66%)
Algerians	02	01 (50%)
Indians	26	19 (73%)
Pakistanis	12	07 (58%)
Bengalis	08	03 (37.5%)
Iranians	06	03 (50%)
Irish	01	01 (100%)

## References

[1] Reveille JD (1998). HLA-B27 and the seronegative spondyloarthropathies. *American Journal of the Medical Sciences*.

[2] Al-Attia HM, Al-Amiri N (1995). HLA-B27 in healthy adults in UAE. An extremely low prevalence in Emirian Arabs. *Scandinavian Journal of Rheumatology*.

[3] Sheth KV, Edwards JA, Godwin JT (1985). Study of the HLA gene and antigen frequency from a Saudi Arabian hospital. *Tissue Antigens*.

[4] Alharbi SA, Mahmoud FF, Al Awadi A, Al Jumma RA, Khodakhast F, Alsulaiman SM (1996). Association of MHC class I with spondyloarthropathies in Kuwait. *European Journal of Immunogenetics*.

[5] Al-Rawi ZS, Al-Shakarchi HA, Hasan F, Thewaini AJ (1978). Ankylosing spondylitis and its association with the histocompatibility antigen HL-A B27: an epidemiological and clinical study. *Rheumatology and Rehabilitation*.

[6] Serre JL, Lefranc G, Loiselet J, Jacquard A (1979). HLA markers in six Lebanese religious subpopulations. *Tissue Antigens*.

[7] Sakly N, Boumiza R, Zrour-Hassen S (2009). HLA-B27 and HLA-B51 determination in Tunisian healthy subjects and patients with suspected ankylosing spondylitis and Behçet’s disease. *Annals of the New York Academy of Sciences*.

[8] Harfouch EI, Al-Cheikh SA (2011). HLA-B27 and its subtypes in Syrian patients with ankylosing spondylitis. *Saudi Medical Journal*.

[9] Al Attia HM, Sherif AM, Moshaddeque Hossain M, Ahmed YH (1998). The demographic and clinical spectrum of Arab versus Asian patients with ankylosing spondylitis in the UAE. *Rheumatology International*.

[10] Al-Arfaj A (1996). Profile of ankylosing spondylitis in Saudi Arabia. *Clinical Rheumatology*.

[11] Tayel MY, Soliman E, El Baz WF, El Labaan A, Hamaad Y, Ahmed MH Registry of the clinical characteristics of spondyloarthritis in a cohort of Egyptian population.

[12] Nazarinia MA, Ghaffarpasand F, Heiran HR, Habibagahi Z (2009). Pattern of ankylosing spondylitis in an Iranian population of 98 patients. *Modern Rheumatology*.

[13] Population structure, Qatar information exchange. http://www.qix.gov.qa.

[14] Askari A, Al-Bdour MD, Saadeh A, Sawalha AH (2000). Ankylosing spondylitis in north Jordan: descriptive and analytical study. *Annals of the Rheumatic Diseases*.

[15] Al-Amayreh IA, Zaidat BO (2000). Ankylosing spondylitis in Northern Jordan. *Saudi Medical Journal*.

[16] Uppal SS, Abraham M, Chowdhury RI, Kumari R, Pathan EM, Al Rashed A (2006). Ankylosing spondylitis and undifferentiated spondyloarthritis in Kuwait: a comparison between Arabs and South Asians. *Clinical Rheumatology*.

[17] Madhavan R, Parthiban M, Panchapakesa Rajendran C, Chandrasekaran AN, Zake L, Sanjeevi CB (2002). HLA class I and class II association with ankylosing spondylitis in a southern Indian population. *Annals of the New York Academy of Sciences*.

